# Simultaneous forecasting of vital sign trajectories in the ICU

**DOI:** 10.1038/s41598-025-99719-w

**Published:** 2025-04-29

**Authors:** Rosemary He, Jeffrey N. Chiang

**Affiliations:** 1 Department of Computer Science, University of California Los Angeles, Los Angeles, CA 90095 USA; 2 Department of Computational Medicine, University of California Los Angeles, Los Angeles, CA 90095 USA; 3 Department of Neurosurgery, University of California Los Angeles, Los Angeles, CA 90095 USA

**Keywords:** Global trajectory predictions, Generative AI, Time series, Clinical support, Health care, Medical research

## Abstract

Individual health trajectory forecasting is a major opportunity for computational methods to integrate with precision healthcare. Recently developed generative AI models have demonstrated promising results in capturing short and long range dependencies in time series data. While these models have also been applied in healthcare, most state-of-the-art are local models, i.e. one model per feature, which is unrealistic in a clinical setting where multiple measures are taken at once. In this work, we extend the framework temporal fusion transformer (TFT), a multi-horizon time series prediction tool, and propose TFT-multi, a global model that can predict multiple vital trajectories simultaneously. We apply TFT-multi to forecast 5 vital signs recorded in the intensive care unit: blood pressure, pulse, SpO2, temperature and respiratory rate. We hypothesize that by jointly predicting these measures, which are often correlated with one another, we can make more accurate predictions, especially in variables with large missingness. We validate our model on the public MIMIC dataset and an independent institutional dataset, and demonstrate our model’s competitive performance and computational efficiency compared to state-of-the-art prediction tools. Furthermore, we perform a study case analysis by applying our pipeline to forecast blood pressure changes in response to actual and hypothetical pressor administration.

## Introduction

In the age of big data, electronic health records (EHR) are paramount in both clinical practice and research^1^. One subset of EHR that has been of interest to both clinicians and researchers is time series data, often measured in laboratory values and vital signs, that can be applied to track disease trajectory from every few minutes to the span of years^2^. Compared to cross-sectional data, which remains constant, time series are more informative of patient development over time. These data can be leveraged to build trajectory prediction tools that can be used for surveillance (e.g., early warning systems^3,4^), and decision support (e.g., forecasting events of interest^5^ or response to interventions^6,7^). Within time series EHR, vital signs are measured most frequently and uniformly for all patients, while other measurements such as laboratory tests can be ordered repeatedly for certain patients. In critical care medicine, it is well established that vital signs including blood pressure, temperature, heart rate, oxygen saturation, and respiratory rate are crucial to monitor as they reflect a patient’s current medical status^8^. Changes in these signs usually indicate abnormal physiological changes which may be associated with complications such as shock, respiratory or cardiac instability, or infection, all of which require timely intervention^9^. As a result, continuous monitoring, early warning and rapid response systems have been employed to detect and address physiological changes as they occur^10,11^. Recent efforts have turned towards the prediction of clinical deterioration of vital signs, particularly in the cases of Covid-19 and sepsis^12^, and towards the forecasting of continuous vital signs to enable proactive response^13–15^.

Successful applications of time series forecasting using generative artificial intelligence (genAI) in finance^16,17^ and climate science^18,19^ have prompted researchers to use AI-based tools to predict trajectories over time in healthcare^20,21^. Compared to traditional autoregressive methods such as the ARIMA model^22^, AI-based models have the potential to better capture complex relationships between variables. In univariate modeling, i.e. predicting one feature at a time, Prophet^23^ is a state-of-the-art method to predict time series data with high seasonality. In 2021, Lim et al. proposed the temporal fusion transformer (TFT)^24^, which allowed the integration of cross-sectional data such as demographics and comorbidities, along with infrequent time series data as covariates to capture both long and short term trends. While these models have been applied for individual forecasting in cardiovascular diseases^25^ and sepsis patients^12^, they are limited by the fact that a separate model and development pipeline must be trained and deployed for each feature. Furthermore, clinical features may be informative of each other and their trajectories should not be predicted independently, prompting the need for global models that can predict multiple variables simultaneously. One of the first multivariate prediction models is the vector autoregressive (VAR) model^26^, which captures the linear interdependencies among multiple time series. In recent years, deep learning models have been applied to multivariate modeling: TSMixer^27^ leverages a multilayer perceptron model, and DeepAR^28^ uses an autoregressive recurrent network to predict multiple time series with covariate inputs. While these models allow for multivariate prediction, they often fail to integrate important covariates or have the unrealistic assumption that all exogenous inputs are known into the future.

In this work, we propose to extend the TFT model from univariate to multivariate prediction and develop an end-to-end pipeline that can simultaneously predict the 5 common vital signs recorded in the intensive care unit (ICU). We demonstrate our method’s comparative performance against state-of-the-art methods in terms of predictive power, as well as its computational efficiency. In addition, we demonstrate a study case to apply our model for treatment effect estimation and decision support, generating hypothetical blood pressure trajectories with and without the administration of pressors. Our pipeline can be extended to real-time predictions and applied as an early warning system for patients in the ICU. We list our contributions as follows:

- We extend TFT from univariate to multivariate modeling to better reflect the clinical setting. Our global forecasting model achieves competitive predictive performance by capturing relations between target variables and requires less computational resources as it predicts multi-horizon trajectories for multiple features all in one run.
- We utilize a masking technique during training to account for losses in the non-imputed values. This allows our model to learn dependencies across real values only and achieve strong performance in validation even in data with large sampling heterogeneity, which is common in time series healthcare data.
- We propose an additional application for our pipeline in treatment effect estimation. We use the model to forecast individual responses to pressors, demonstrating the ability to compare alternative trajectories in hypothetical scenarios to supplement clinical decision support.

## Methods

### Data source

This study was performed using two independent datasets. Model development leveraged the publicly available MIMIC-IV v2.2^29,30^ dataset. MIMIC-IV is an EHR dataset consisting of patients at the Beth Israel Deaconess Medical Center. A second dataset consisting of de-identified electronic health records were extracted from a large academic medical center for external model validation. The study design consisted of retrospective analysis using de-identified data and was thus deemed non human-subjects research by the local IRB. Informed consent and ethics approval are waived by the approval of local IRB. The study was conducted in accordance with relevant guidelines and regulations.

### Data preparation

We train and validate our model using the MIMIC dataset and perform external validation on the second private institutional dataset. After initial filtering, the MIMIC set consists of 9,638 patients who were admitted to the intensive care unit (ICU). The validation institutional cohort consists of 35,286 patients who presented at the Emergency Department and were eventually admitted to the ICU. We summarize some key characteristics of both cohorts in Table 1.

**Table 1:** Cohort characteristics of MIMIC (train/test) and private institutional (external validation) dataset, summarized by mean±standard deviation or percentage indicated by $\%$.

Sample characteristics	MIMIC (n=9638)	Institutional (n=35286)
Length of stay (days)	3.57±0.25	14±22
Demographics		
Male (%)	45.6	43.2
Age	62±16	55±23
Ethnicity (%)		
Caucasian	68	42
African American	10	11
Asian, Pacific Islander	3.1	10
Native American	0.2	0.2
Comorbidities (%)		
Asthma	13.1	6.7
Diabetes	34.3	17.8
Heart failure	32.1	7.6
Medication (%)		
Pressors	30	30

We include static and time series, numerical and binary variables including 5 demographic variables such as age and BMI, 72 comorbidities including heart failure and diabetes, 21 laboratory results including blood glucose and potassium, 12 medications in the class of pressors including Epinephrine and Angiotensin, to predict 5 common vital signs: mean arterial blood pressure (mean BP), pulse, SpO2, respiratory rate (Resp) and temperature (Temp). We resample all time series data into 15 minute intervals and aggregate using the mean (for numeric variables) or median (for categorical variables) within each interval, and then forward-fill for any missing intervals (similar to past approaches^5^). For static variables, we fill missing values with 0 for binary inputs such as comorbidities and drop samples with missing numeric values. For medications, we group all pressor administrations into one categorical variable and assume a missing value indicates no medication given. For each encounter, we construct 100 time points and use the first 75 as the past (18.75 hours) and the last 25 as the future (6.25 hours). During model training, different variables are considered as follows: demographics and comorbidities as past static variables; laboratory results, vital signs, age and medications up until the first 75 time points as past time series variables; age in the last 25 time points as known future time series variables. We randomly split the MIMIC cohort by individuals to 80% training set and 20% held-out test set.

### Temporal fusion transformer

In this section, we introduce temporal fusion transformer (TFT)^24^, a transformer based model for multi-horizon time series forecasting. TFT introduces a novel strategy to incorporate static and time series covariates separately, preserving both short and long range dependencies between variables. There are several building blocks that make TFT powerful in time series prediction. First, historical time series inputs are passed through sequence-to-sequence layers to generate context vectors for short range dependencies. Meanwhile, static covariates are passed through an encoder to generate a representation, which is then combined with the context vectors in the static enrichment layer. Next, these embeddings are fed into multi-head attention blocks, which are better at capturing long term relations^31^. Lastly, the output from the attention layer is processed by more densely connected layers to produce prediction forecasts. In multiple parts of the architecture, variable selection units are incorporated to select relevant covariates to pass onto the next layer. TFT allows different data types to be incorporated at different points in the model: past static variables, past time series variables, and future time series variables. The model outputs multi-horizon forecasts for 3 quantiles: the 10th, 50th and 90th. While the 50th quantile is often closest to the ground truth, the 10th and 90th quantiles act as prediction upper and lower bounds. By incorporating and processing different types of covariates and combining strengths from short and long term dependencies, TFT is a state-of-the-art method in predicting electricity, traffic and stock market patterns^24^. In addition, feature weights can be extracted across multi-head attention layers for interpretability. In other studies, TFT have shown comparable results in vital prediction^12^, prompting our work to extend it to simultaneous prediction for multiple variables in sparsely sampled data.

**Fig. 1:**
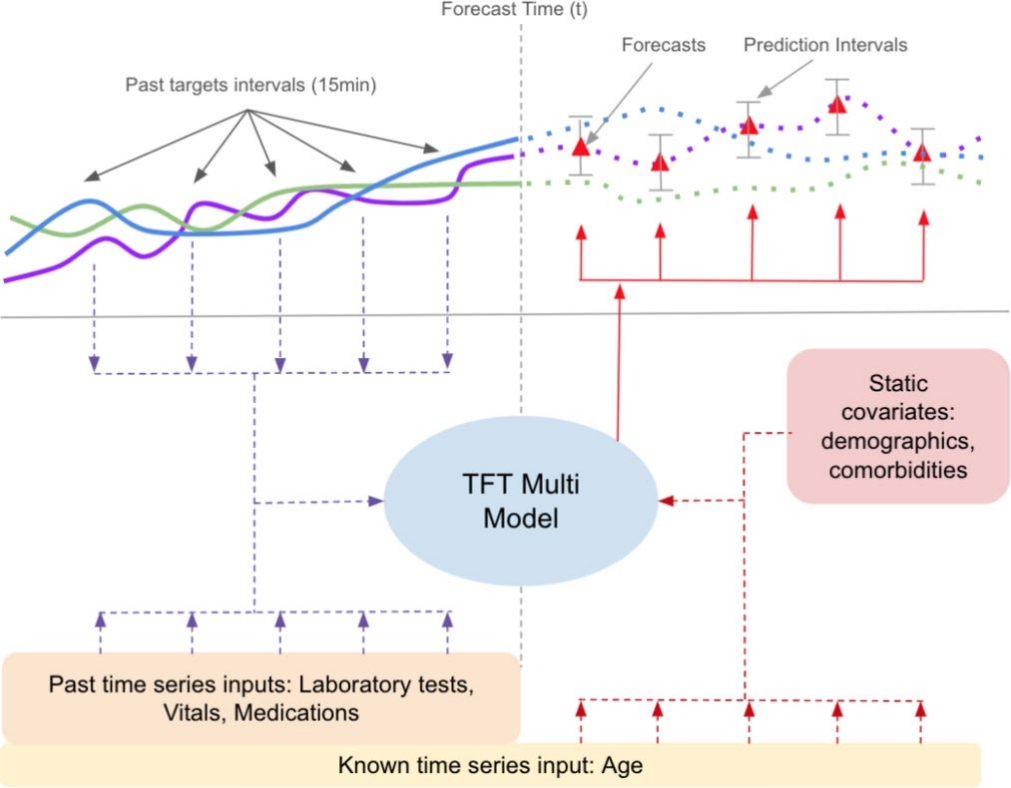
Model workflow for simultaneously predicting 3 example variables.

### TFT-multi

We propose TFT-multi, our extension to the original model, that is capable of handling sparsely sampled data and simultaneously predicting multiple measures of interest (in this work 5 vital signs in ICU monitoring). In Fig. 1, we show an illustration of our end-to-end pipeline that takes in time series and static covariates and simultaneously predicts 3 toy trajectories.

We modify TFT in two main ways: the input-output structure and the loss function, while keeping the inner network the same. In the original model, past target values are appended to the past time series covariates, which we now extend from one to multiple at each time point. In the output, instead of predicting three quantiles at each time point for one target, we modify the last fully connected layers to output three quantiles for each target variable all at once. We make two modifications in the loss function. First, we sum over trajectory losses over all quantiles and variables to jointly optimize all target variables. Second, we apply a masking technique to only account for losses in the real values and not the imputed ones to avoid overfitting to imputed data in sparsely sampled variables and increase predictive performance. Our loss function is as follows:

1 $$\begin{aligned} \mathcal {L}(\Omega ) = \sum _{v \in V} \sum _{y_i \in \Omega } \sum _{q \in \mathbb {Q}} \frac{\sum _{t = 1}^{t_{\text {max}}} QL(y_{it}^v, \hat{y}_{it}^v(q), q) \cdot \mathbb {I}_{y_{it}^v}({\text {real value}})}{\sum _{t = 1}^{t_{\text {max}}}\mathbb {I}_{y_{it}^v}({\text {real value}})} \end{aligned}$$

2 $$\begin{aligned} QL(y, \hat{y}, q) = q * \text {max}(0, (y - \hat{y})) + (1 - q) * \text {max}(0, (\hat{y} - y)), \end{aligned}$$

where *V* is the set of target variables, $\Omega$ is the training dataset, *Q* is the set of quantiles $\{0.1,0.5,0.9\}$ across future time points *t* to $t_\text {max}$. We use the notation $\mathbb {I}(x)$ as the indicator function with value 1 when *x* is true and 0 otherwise.

### Model training

We trained our model using the Adam^32^ optimizer with a learning rate of 1e-3, a batch size of 800, a dropout rate of 0.3, state size of 240 and 2 attention heads. All hyperparameters were optimally selected from hyperparameter tuning with the following combinations: learning rate {1e-3, 1e-4, 1e-5}, batch size {600, 800, 1000}, dropout rate: {0.1, 0.3, 0.6}, state size {120, 240}, and attention head {2,4}. We trained the model for a maximum of 2000 epochs and implemented early stopping after the loss increases continuously for 20 epochs. We ran the model in parallelization across two Tesla V100-PCIE-16GB GPUs, and training took 6 hours. For testing, we ran inference on the held-out test set and the external validation set and calculate the mean absolute error and mean absolute percentage error, shown in Table 2.

### Reference models

We chose 5 state-of-the-art univariate and multivariate models for comparison: Prophet^23^, VAR^26^,TSMixer^27^, deepAR^28^, the original TFT^24^. For univariate models, Prophet leverages an additive regression model with growth trends and TFT is transformer based. For multivariate models, VAR is a vector autoregressive model, TSMixer is a multilayer perceptron with time and feature mixing, and deepAR is an autoregressive neural network. We used the same training, validation, and external test sets across all model training and validation. For univariate forecasting models, we fitted a separate instance to predict each of the five vital signs. For Prophet^23^, we trained with default parameters and change point scale of 0.01, and excluded covariates not known in the future since Prophet requires all covariate inputs to be known into the future. For TFT^24^, we trained with the same inputs, hyperparameter choices and procedure as our model. For VAR^26^, we fitted the model to take in past vital sign trajectories and no exogenous variables, in accordance with convention. We note here that respiratory rate and temperature did not pass the Granger’s causality test^33^, which determines if one time series can predict another time series, we did not include them in the model^26^. By the same logic, we did not include temperature in the external validation set. For both TSMixer^27^ and deepAR^28^, we included the same covariates and trained both models on the same training set for 1000 epochs or until convergence. We used the same learning rate of 1e-3 and a batch size of 800 as our model, and the default choice for other hyperparameters.

### Treatment response forecasting

We conduct a study case applying our prediction pipeline to forecast patient trajectories conditioning on whether a patient is administered pressors. Pressors (vasopressors) are a class of drugs which raise blood pressure and are used in the management of hypotension and shock^34^. They are clinically indicated when mean blood pressure falls below 60 mmHg. As these drugs are expected to increase blood pressure within a short time, blood pressure forecasts should reflect this trend when provided time-varying medication administration. In this analysis, we use the MIMIC cohort only, as the institutional dataset obtained does not have enough information about the injection time and duration of medications. Our experimental set-up is as follows. First, we train a separate TFT-multi model assuming that medication (pressor) administration is known into the future, i.e. as a future time series variable. Then for each patient in the test set, we make forecasts based on three scenarios: the observed truth for medication input, assuming constant administration throughout the prediction interval (all 1s), and assuming no administration (all 0s).

## Results

We hypothesize that leveraging a global model to predicts multiple features at once gives us two main advantages: better performance in undersampled features and computational efficiency. We compare performance in predicting 5 standard vital values in the ICU: mean arterial blood pressure (mean BP), pulse, temperature (temp), respiratory rate (resp) and SpO2 between our model against the 5 reference models. We used the mean absolute error (MAE) as the comparison metric in both validation datasets across all vital predictions. We examined only the time points with recorded and not imputed values, calculated as follows for each vital sign:

3 $$\begin{aligned} MAE = \frac{\sum _{y_i \in \Omega } \sum _{t = 1}^{t_{\text {max}}} |y_{it} - \hat{y}_{it}| \cdot \mathbb {I}_t({\text {real value}})}{\sum _{y_i \in \Omega } \sum _{t = 1}^{t_{\text {max}}} \mathbb {I}_t({\text {real value}})} \end{aligned}$$

### Trajectory prediction

We compare performances for each vital sign, with the best performing model in bold in Table 2. The top 6 rows are calculated with the held-out test set, while the bottom 6 with the external validation (EV) set. For TFT and TFT-multi, we take the 50th percentile prediction as the prediction. In the held-out test set, TFT-multi archives the lowest MAE in meanBP, pulse and temperature, and is a close second to the best performing model for SpO2 (Prophet) and respiratory rate (TSMixer). In external validation, TFT-multi achieves the lowest MAE for 3 out of 5 vitals, and performs comparably with Prophet in SpO2 and TSMixer in temperature. Compared to the original TFT model, there is a significant improvement in predicting vitals that have more missingness, such as SpO2.

**Table 2:** Model performance across five forecasted vital signs.

	Mean BP	Pulse	SpO2	Resp	Temp
Prophet (test)	13.4 [15.6]	10.9 [11.2]	**1.7 [1.9]**	5.6 [16.2]	3.6 [3.9]
VAR	>100 [>1]	>100 [>1]	>100 [>1]	N/A	N/A
TSMixer	10.41[12.25]	11.06[13.71]	2.77[3.43]	**1.15[8.12]**	0.8[0.83]
deepAR	13.86 [16.06]	13.72 [16.59]	2.03 [2.83]	4.5 [31.69]	0.76 [0.77]
TFT	11.9 [12]	13.4 [13.2]	14.4 [15]	4 [12.6]	1.3 [1.3]
TFT-multi (ours)	**7.4 [8.1]**	**9 [8.9]**	1.8 [2]	4.5 [13]	**0.7 [1]**
Prophet (EV)	8.2 [11]	9.3 [11]	**1.7 [2.8]**	3 [41]	0.9 [0.9]
VAR	6.5 [9.7]	7.2 [8.6]	>100 [>1]	2.5 [51.4]	N/A
TSMixer	8.87 [9.96]	9.57 [12.02]	12.75 [13.38]	2.91 [13.71]	**0.66 [0.69]**
deepAR	12.61 [12.73]	13.95 [17.38]	1.93 [2.14]	3.69 [20.14]	0.74 [0.77]
TFT	10.1 [12.4]	8.7 [10.1]	17.09 [22.8]	2.42 [32.2]	1.4 [1.4]
TFT-multi (ours)	**6.2 [8.9]**	**7.2 [7.8]**	1.8 [2.8]	**2.4 [30.1]**	0.8 [0.7]

Values indicate mean absolute error [mean absolute percentage error(%)] for the held-out test and external validation sets. Best performance for each vital sign in bold.

**Fig. 2:**
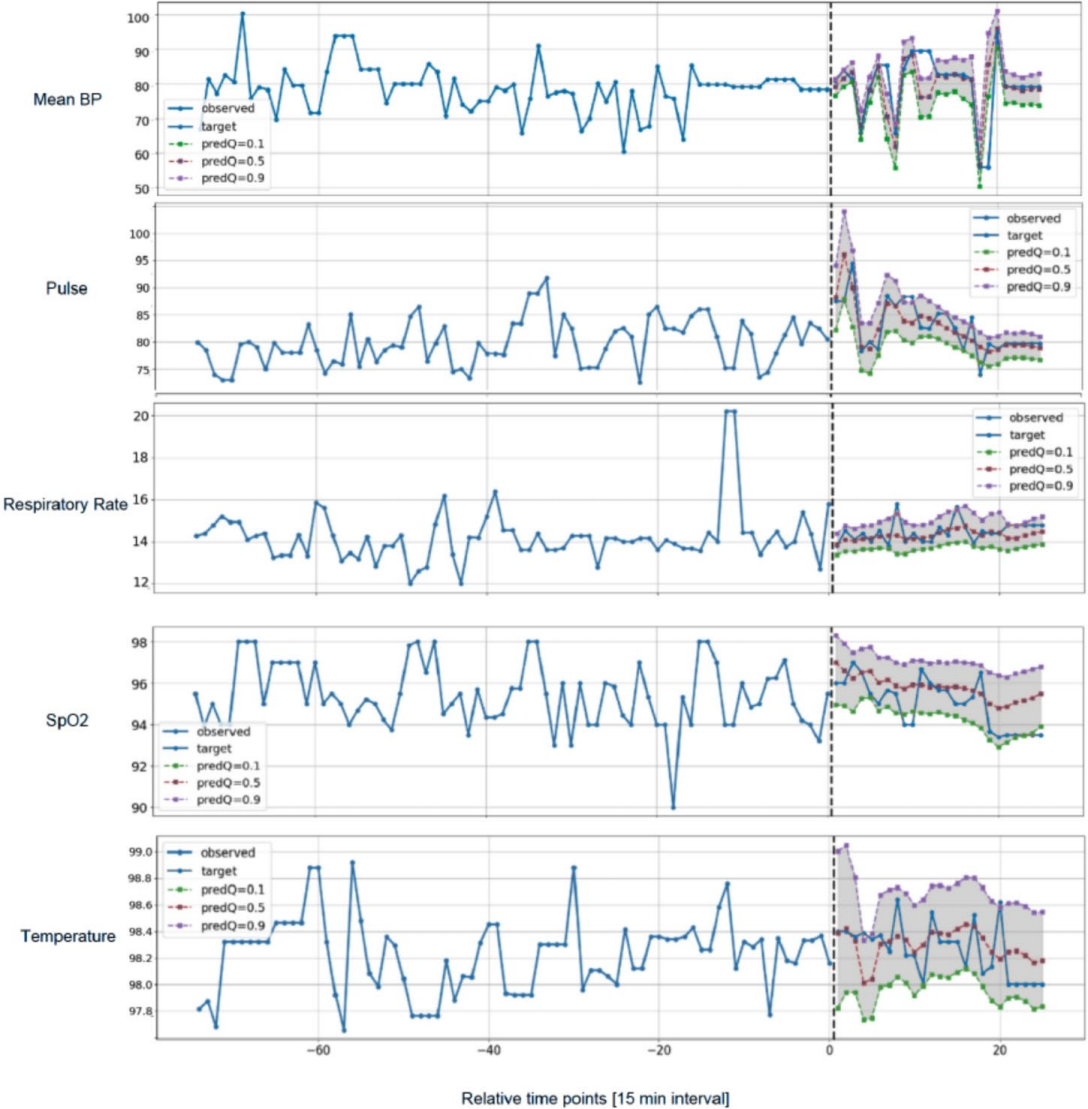
Sample prediction visualization across vital features. Different colors indicate different trajectories: blue indicates observed historical data; orange indicates ground truth; green, red and purple indicate the 10th, 50th and 90th percentile prediction respectively.

**Fig. 3:**
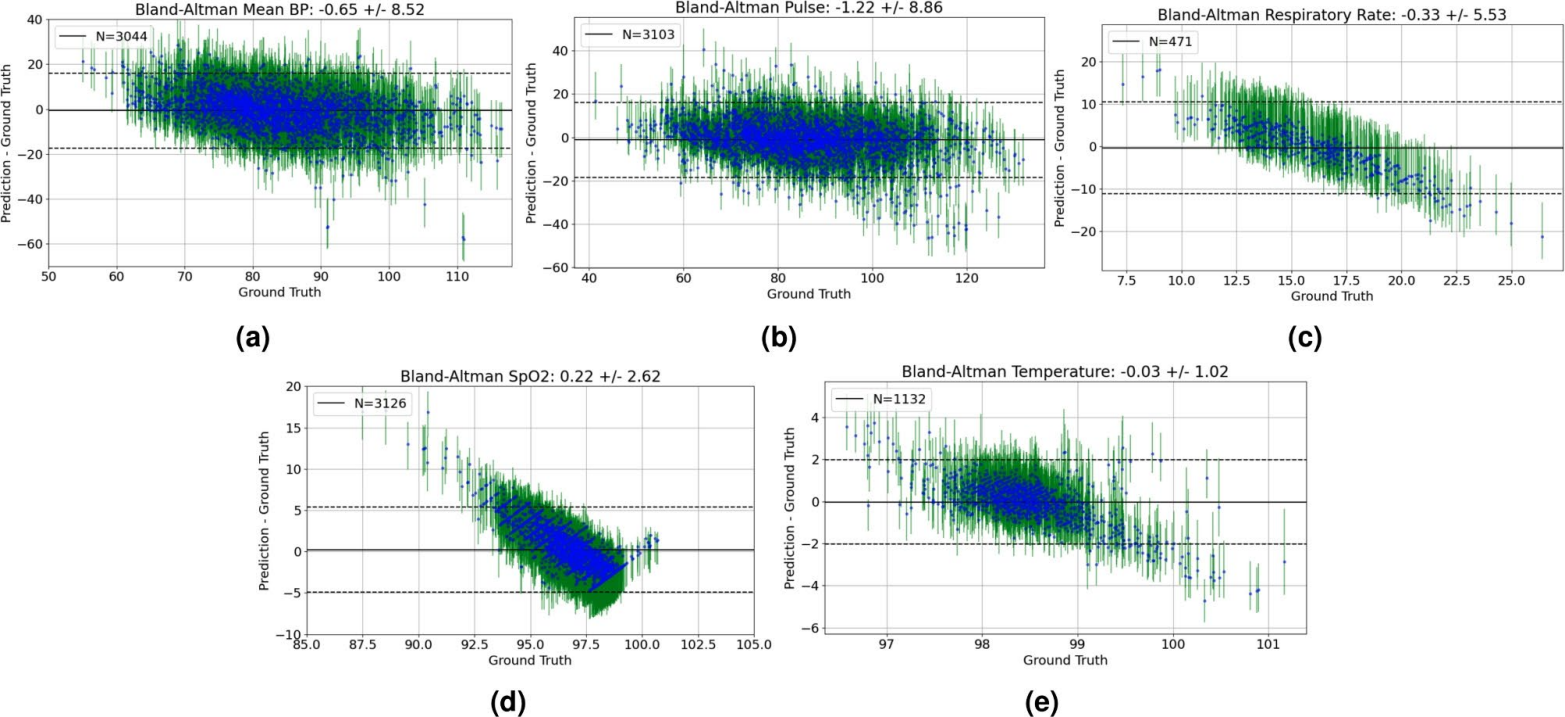
Bland-Altman Plots for predictions in the held-out test set across vitals. Each point represents an observed time point. Vertical bars represent the upper and lower prediction bound errors; solid line indicate the mean difference, dashed lines indicate the mean difference $\pm 1.96$ times the standard deviation of differences.

In Fig. 2, we show a visualization of a sample prediction of our model from the external validation set. For mean BP and pulse, the 50th percentile follows closely to the true values and fluctuations. In vitals with more missingness such as SpO2 and temperature, TFT-multi estimates a reasonable bound but does not track fluctuations well, which prompted additional analysis to examine the calibration of our model on top of MAE, which may be biased by the fact that the majority of measurements tend to fall within the “normal” range. In Fig. 3, we show the calibration of TFT-multi’s prediction at the 50th percentile with the ground truth in the test set across all vitals using a Bland-Altman plot^35,36^. While TFT-multi appears to be well calibrated for mean BP and Pulse, we observe limited calibration for respiratory rate, SpO2, and temperature despite low MAE values, prompting further investigation.

#### Prediction bound estimation

**Fig. 4:**
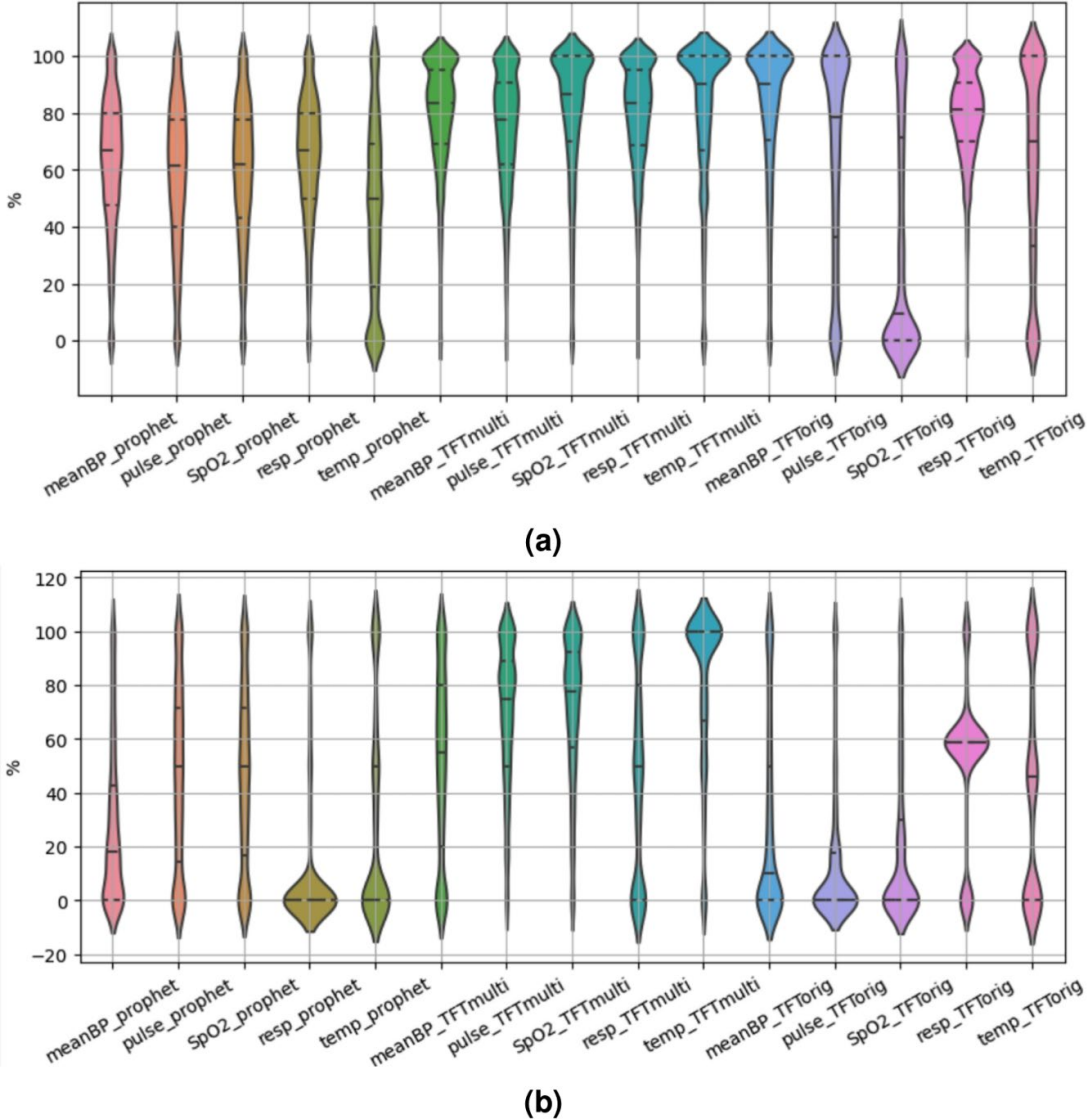
Violin plot comparing percentage of true trajectory within the estimated bounds across vitals of interest for three methods: prophet, TFT-multi (ours) and TFT. Lines within each plot represent the first, second (median) and third quartile values of the held-out test set in (**a**) and external validation set in (**b**).

In addition to exact values, prediction bounds are sometimes helpful as vital measures have high interpersonal differences and a value is considered abnormal only if it falls outside a range. Therefore, we performed an additional analysis to better understand model performances in predicting upper and lower bounds in models that output prediction bounds. For each subject in both validation sets, we calculated the percentage of times a true value is within prediction bounds. For Prophet, we used y_hat_upper and y_hat_lower as bounds, which capture uncertainty in trend, seasonality and observation noise in the prediction^23^. For TFT^24^ and TFT-multi, we used the 10th and 90th quantile predictions. In Fig. 4a , we show the percentage distribution over the test set for all 5 vital signs in a violin plot and the first, second and third quartile values as lines within each plot. In addition to having the lowest MAE, TFT-multi’s prediction bounds better capture forecasted trajectories than Prophet and TFT. Consistent with the Bland-Altman analysis (Fig. 3), our findings suggest that for values that have high missingness such as SpO2, metric calculation using only real values may not be the best as there are few values. In Fig. 4b , we show results for the external set, where performances across methods have decreased as expected, but TFT-multi still remains the best performing method.

### Interpretability analysis

**Fig. 5:**
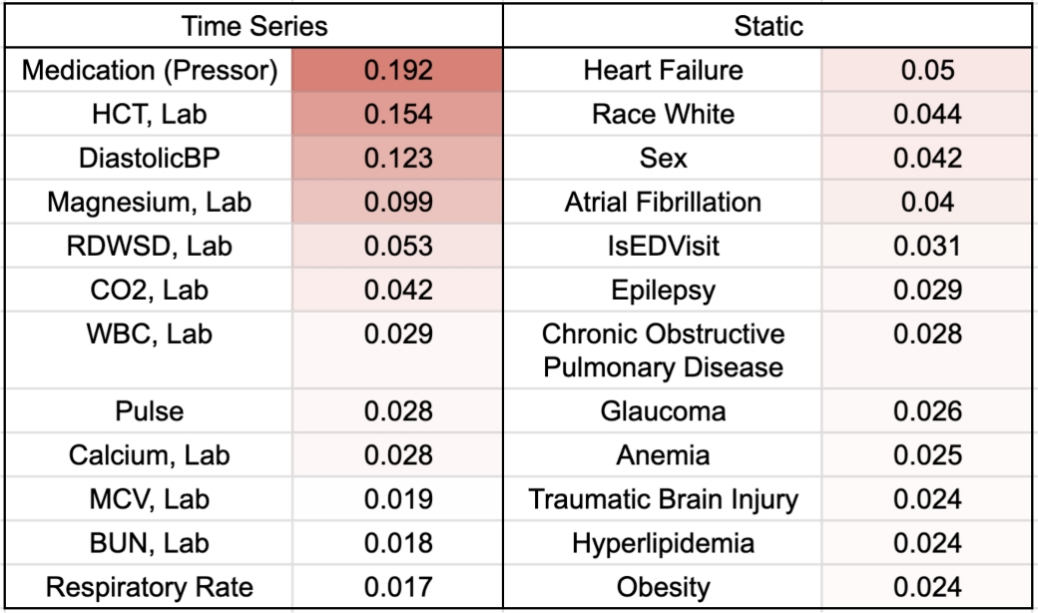
Top features by importance in predicting the 50th percentile trajectory for both static and time series variables. Color gradient reflects importance by weight.

We conducted a feature importance test, as proposed in the original TFT paper^24^, by aggregating the multi-head attention weights for each input feature to see which input had the most influence over the predictions. We show the top features by weight for both the time series and static inputs in Fig. 5. For time series inputs, medication has the most influence over vital predictions. This aligns with our expectation, as pressors should influence blood pressure significantly within a short period of time. Among static variables, comorbidities such as heart failure, arterial fibrillation and chronic obstructive pulmonary disease have the most effects over vital signs. We note here the feature importance is reported across all patients and time points, which may not be informative on a subject level and is a shortcoming of this analysis. For each subject, the factors that affect their prediction most may be different and will be studied in future works.

### Impact analysis of historical data length

To evaluate the effect of historical data length, we ran additional experiments by comparing model performance between three “lookback” window lengths: 3, 9 and 18.75 hours, and compare results in Table 3. While the three options performed comparably, a larger “lookback” window generally leads to better predictive performance in most measures in the testing set and all measures in the validation set. Interestingly, even with the smallest window size (3-hr), TFT-multi outperforms other methods in Table 2 that had more historical data in measures with a large missing rate. We suspect this is due to our hypothesis that a global prediction model will outperform several local models by learning overall trends, especially in vitals with large missingness.

**Table 3:** Model performance across vital signs using varying length of historical data.

Window length	Mean BP	Pulse	SpO2	Resp	Temp
3-hr (test)	10.1 [11.1]	8.6 [10]	1.8 [2.1]	2.7 [13.9]	0.8 [0.8]
9-hr	8.6 [9.8]	**8 [9.8]**	1.8 [2.1]	**2.6 [13.9]**	0.8 [0.8]
18.75-hr	**7.4 [8.1]**	9 [8.9]	**1.8 [2]**	4.5 [13]	**0.7 [1]**
3-hr (EV)	10 [11.8]	7.6 [9]	2.3 [2.5]	3.9 [24.4]	0.85 [0.8]
9-hr	9.9 [11.4]	7.5 [9.2]	2.7 [2.8]	3.4 [24.4]	0.95 [1]
18.75-hr	**6.2 [8.9]**	**7.2 [7.8]**	**1.8 [2.8]**	**2.4 [30.1]**	**0.8 [0.7]**

Values indicate mean absolute error [mean absolute percentage value(%)] for held-out test and external validation set. Best performance for each vital sign in bold.

**Table 4:** T-test trajectory comparison for forecasting mean blood pressure conditioned on medication (pressor) intake.

Hypothesis	Statistic	P-value
mean($\hat{y}|$medication=1) - mean($\hat{y}|$medication=0) = 0	3.67	2e-4
mean($\hat{y}|$medication=1, obs=1) - mean($\hat{y}|$medication=0, obs=1) = 0	3.12	1e-3
mean($\hat{y}|$medication=1, obs=0) - mean($\hat{y}|$medication=0, obs=0) = 0	2.19	0.028

### Treatment response forecasting

Lastly, we extend our model’s application beyond forecasting and conducted a study case in medication effect estimation. For patients who received pressor administrations within the course of their ICU care, we forecasted three trajectories: (1) assuming the true administration; (2) assuming continuous administration (all 1’s); and (3) assuming no administration (all 0’s). First, we calculated a MAE for real points between these scenarios and report the results: 7.44 when the ground truth pattern is used, 7.47 when setting pressor administration to 1, and 7.46 when setting pressor administration to 0. As expected, the actual medication input pattern produces predictions most closely fit with observation than setting the medication to all 1s or 0s. Second, we applied t-tests to test that the modeled effect of pressor administration was consistent with its intended effect. We present our t-test results in Table 4, where for $\hat{y}$ we use the 50th percentile prediction. We performed 3 comparison tests, the first in which we compared the mean trajectory prediction between the two hypothetical scenarios without considering the ground truth. In row 1, we see an average increase of 3.67 in blood pressure when assuming some medication is administered. The second and third comparisons took real observations into account and tested for predicted differences between scenarios for time points that have a ground truth observation (obs) of 1 or 0. When the ground truth observation is no medication (obs=0), hypothetically modeling pressor administration predicts a value 3.12 higher than the alternative. When the patient was given medication in real life, the difference is 2.19. In all three scenarios, the p-values are significant ($<0.05$) to reject the null hypothesis and could indicate that our model is learning the association between increased BP and pressor administration.

## Discussion

In this work, we extended TFT, a univariate forecasting model, to TFT-multi, a global model that simultaneously predicts 5 vital signs for patients in the ICU. We highlight two main advantages of our model: improvement in predictive power in undersampled features and computational efficiency. While the original TFT had difficulty predicting variable trajectories with high missingness such as SpO2 by itself, TFT-multi uses its relation with other vitals such as pulse to improve prediction. Prophet’s strong performance in SpO2 is expected, as it is highly regular and cyclical. TSMixer showed strong ability to predict respiratory rate and temperature but not the other vital signs, suggesting it may also be sensitive to sampling heterogeneity. With the same training sample size, TFT and TFT-multi both took 6 hours to train over 2000 epochs, Prophet took 1 hour to fit a univariate model, VAR took 0.5 hour, TSMixer took 2 hours and deepAR took 6 hours to train over 1000 epochs. A clear advantage for multivariate models is computational speed. For TFT and Prophet, to predict all vitals would require training 5 models over 30 and 6 hours, respectively.

Next, we discuss some limitations and future directions. First, the model has shown limited calibration in predicting features with high missingness and difficulty tracking frequent fluctuations in some cases. We hypothesize a contributing factor to be the data itself: respiratory rate has a lower sampling rate and may not provide enough data for the model to fully learn its pattern (as observed in Fig. 3a); SpO2 and temperature have a limited range of observations in our training data, as observed in Fig. 3b and 3c . Second, interpretability in generative models has been a long-standing issue^37^. While we try to interpret the model by analyzing aggregated attention weights, further work can be done to analyze feature importance on a subject-level. Lastly, in our proof-of-concept medication analysis, we assumed a binary administration of pressors while in reality medications are given intermittently and at varying doses. Future work will include modeling these medication inputs with more variability to test performance in a more realistic setting.

Our pipeline has the potential to be incorporated into clinical practice in several ways. In this work, we demonstrated one use case of our pipeline for treatment effect estimation, which can be extended into a counterfactual estimation tool. It can also be extended into an all-in-one real time early warning system, which can assist clinicians in monitoring ICU patients and predict anomalous events up to 6 hours prior. Furthermore, the model can be adapted to other data modalities including medical images and text data, which are highly complex and often serial in nature. Another direction for this work is to extend it to the outpatient cohort, which is more representative of the population but has large amounts of missing in time series data. A model to first impute irregularly sampled time series data can be added in the pipeline to address this issue and predicts longer range trajectories in the outpatient cohort, benefiting a larger population. By adapting powerful AI methods to better address the unique challenges in EHR and making them easier to use for users with little computer science background, our work has the potential to improve patient care in a real world setting.

## Data Availability

The validation dataset is not publicly available due to institutional policy. The MIMIC training dataset is publicly available and published in paper, the implementation of TFT-multi is available for reproducibility and can be found at the GitHub repository https://github.com/rosie068/TFT-multi. Please contact the corresponding author for data-related requests.
